# Transcriptomic analysis of longitudinal *Burkholderia pseudomallei* infecting the cystic fibrosis lung

**DOI:** 10.1099/mgen.0.000194

**Published:** 2018-07-10

**Authors:** Erin P. Price, Linda T. Viberg, Timothy J. Kidd, Scott C. Bell, Bart J. Currie, Derek S. Sarovich

**Affiliations:** ^1^​ Faculty of Science, Health, Education and Engineering, University of the Sunshine Coast, Sippy Downs, QLD, Australia; ^2^​ Global and Tropical Health Division, Menzies School of Health Research, Charles Darwin University, Darwin, Northern Territory, Australia; ^3^​ Faculty of Medicine, The University of Queensland, Brisbane, QLD, Australia; ^4^​ School of Chemistry and Molecular Biosciences, The University of Queensland, St Lucia, QLD, Australia; ^5^​ QIMR Berghofer Medical Research Institute, Herston, QLD, Australia; ^6^​ Department of Thoracic Medicine, The Prince Charles Hospital, Chermside, QLD, Australia; ^7^​ Department of Infectious Diseases and Northern Territory Medical Program, Royal Darwin Hospital, Darwin, Northern Territory, Australia

**Keywords:** bacterial transcriptomics, convergence, RNA-seq, evolution, antibiotic resistance, pathoadaptation

## Abstract

The melioidosis bacterium, *Burkholderia pseudomallei*, is increasingly being recognised as a pathogen in patients with cystic fibrosis (CF). We have recently catalogued genome-wide variation of paired, isogenic *B. pseudomallei* isolates from seven Australasian CF cases, which were collected between 4 and 55 months apart. Here, we extend this investigation by documenting the transcriptomic changes in *B. pseudomallei* in five cases. Following growth in an artificial CF sputum medium, four of the five paired isolates exhibited significant differential gene expression (DE) that affected between 32 and 792 genes. The greatest number of DE events was observed between the strains from patient CF9, consistent with the hypermutator status of the latter strain, which is deficient in the DNA mismatch repair protein MutS. Two patient isolates harboured duplications that concomitantly increased expression of the β-lactamase-encoding gene *penA*, and a 35 kb deletion in another abolished expression of 29 genes. Convergent expression profiles in the chronically-adapted isolates identified two significantly downregulated and 17 significantly upregulated loci, including the resistance-nodulation-division (RND) efflux pump BpeEF–OprC, the quorum-sensing *hhqABCDE* operon, and a cyanide- and pyocyanin-insensitive cytochrome *bd* quinol oxidase. These convergent pathoadaptations lead to increased expression of pathways that may suppress competing bacterial and fungal pathogens, and that enhance survival in oxygen-restricted environments, the latter of which may render conventional antibiotics less effective *in vivo*. Treating chronically adapted *B. pseudomallei* infections with antibiotics designed to target anaerobic infections, such as the nitroimidazole class of antibiotics, may significantly improve pathogen eradication attempts by exploiting this Achilles heel.

## Data Summary

1. Whole-transcriptome sequence data have been deposited into the National Center for Biotechnology Information (NCBI) Sequence Read Archive (SRA) under BioProject accession PRJNA398168 (url – https://www.ncbi.nlm.nih.gov/bioproject/PRJNA398168).

2. The SRA accession number for the raw transcriptomic data of MSHR5654 is SRR6031143 (https://www.ncbi.nlm.nih.gov/sra/?term=SRR6031143).

3. The SRA accession number for the raw transcriptomic data of MSHR5651 is SRR6031144 (https://www.ncbi.nlm.nih.gov/sra/?term=SRR6031144).

4. The SRA accession number for the raw transcriptomic data of MSHR5670 is SRR6031145 (https://www.ncbi.nlm.nih.gov/sra/?term=SRR6031145).

5. The SRA accession number for the raw transcriptomic data of MSHR5662 is SRR6031146 (https://www.ncbi.nlm.nih.gov/sra/?term=SRR6031146).

6. The SRA accession number for the raw transcriptomic data of MSHR8437 is SRR6031147 (https://www.ncbi.nlm.nih.gov/sra/?term=SRR6031147).

7. The SRA accession number for the raw transcriptomic data of MSHR8436 is SRR6031148 (https://www.ncbi.nlm.nih.gov/sra/?term=SRR6031148).

8. The SRA accession number for the raw transcriptomic data of MSHR8440 is SRR6031149 (https://www.ncbi.nlm.nih.gov/sra/?term=SRR6031149).

9. The SRA accession number for the raw transcriptomic data of MSHR8438 is SRR6031150 (https://www.ncbi.nlm.nih.gov/sra/?term=SRR6031150).

10. The SRA accession number for the raw transcriptomic data of MSHR8442 is SRR6031151 (https://www.ncbi.nlm.nih.gov/sra/?term=SRR6031151).

11. The SRA accession number for the raw transcriptomic data of MSHR8441 is SRR6031152 (https://www.ncbi.nlm.nih.gov/sra/?term=SRR6031152).

**Impact Statement**

The melioidosis bacterium *Burkholderia pseudomallei* is a rare but important pathogen in cystic fibrosis (CF). *B. pseudomallei* infection of the CF airways commonly shifts towards a chronic state, where it becomes difficult to eradicate, leading to accelerated lung decline, respiratory failure and death. Understanding pathogen adaptation to the CF airways is essential for identifying targeted treatment regimens that can successfully slow or halt disease progression. We have recently catalogued the genome-wide evolution of *B. pseudomallei* in the lungs of seven CF melioidosis patients, which revealed several important adaptations conferring antibiotic resistance, virulence factor attenuation and genetic loss. Here, we extend upon our earlier study by characterising paired *B. pseudomallei* transcriptomes from five cases using RNA sequencing (RNA-seq). Differential gene expression was identified in four of the five cases, and affected loci involved in antibiotic resistance, quorum sensing, adaptation to microaerobic conditions and inhibition of competing pathogen species. Notably, RNA-seq identified additional within-host evolution signatures not observable with genomic data. Our results provide evidence for parallel evolution of *B. pseudomallei* in the CF lung, a trait that may be exploited for targeted pathogen eradication and improved patient outcomes.

## Introduction

The Gram-negative soil-dwelling bacterium *Burkholderia pseudomallei* causes melioidosis, an opportunistic tropical infectious disease of humans and animals that has a high fatality rate [1]. *B. pseudomallei* is found in many tropical and subtropical regions globally, and has been unmasked in temperate and even arid environments following unusually wet weather events [2–4]. Infection occurs following percutaneous inoculation from contaminated soil or water, inhalation, or ingestion. Melioidosis symptoms vary widely due to the ability of *B. pseudomallei* to infect almost any organ, with pneumonia being the most common presentation [5, 6]. Individuals most at risk of contracting melioidosis include diabetics, those with hazardous alcohol consumption and the immunosuppressed. There has been increasing recognition that people with chronic lung diseases such as cystic fibrosis (CF) are also at a heightened risk [7, 8].

CF is a heritable disorder of the *CFTR* gene, and defects in CFTR (cystic fibrosis transmembrane conductance regulator) lead to exaggerated airway inflammation, an imbalance in salt regulation in the lungs and pancreas, and a chronic overproduction of thick and sticky mucus in the airways and digestive system [9]. Impaired immunity and mucus clearance encourage infection and subsequent persistence and adaptation of opportunistic bacterial pathogens in the CF lung, leading to the development of bronchiectasis with subsequent progressive pulmonary decline, and ultimately, loss of pulmonary function and death [10].

The most common pathogens of the CF lung are *Pseudomonas aeruginosa*, *Staphylococcus aureus*, *Haemophilus influenzae*, and less commonly, *Achromobacter xylosoxidans*, non-tuberculosis mycobacteria, *Stenotrophomonas maltophilia* and *Burkholderia cepacia* complex species [11]. The most common and best-studied CF pathogen is *P. aeruginosa*, which can adapt to the CF lung environment via various mechanisms. Convergent pathoadaptations in *P. aeruginosa* include the downregulation or loss of virulence factors and motility-encoding loci, emergence of hypermutators, enhanced antibiotic resistance and immune evasion facilitated by a switch to mucoidy and a biofilm-based lifestyle, and altered expression of other loci enhancing bacterial metabolism and survival within the nutrient-poor CF lung environment [10, 12].

Improving life expectancy for those with CF has led to an increased risk of exposure to *B. pseudomallei* following travel to melioidosis-endemic regions. Although uncommon, infection of the CF lung by *B. pseudomallei* has now been documented in at least 25 cases worldwide [13]. Due to low total case numbers, comparatively little is understood about the pathogenic role of *B. pseudomallei* in CF pulmonary disease. The most common clinical presentation is chronic infection (76 %), which is associated with accelerated lung function decline [13]. This prevalence contrasts with melioidosis in non-CF patients, where chronic infection occurs in only 11 % of cases [6]. To better understand *B. pseudomallei* pathoadaptation in the CF lung, we recently investigated the genome-wide evolution of isogenic *B. pseudomallei* strains isolated from seven Australasian CF patients, which were collected between 4 and 55 months apart [14]. Hallmarks of these infections included *B. pseudomallei* persistence despite multiple eradication attempts, multidrug resistance, mutations in virulence, metabolism and cell wall components, and the first-documented case of hypermutation in *B. pseudomallei*. In all except one case, multiple single-nucleotide polymorphism (SNP) and insertion-deletion (indel) mutations were identified, with a high rate of nonsynonymous mutations, many of which were predicted to affect protein function [14].

RNA sequencing (RNA-seq) provides a detailed view of the transcriptional landscape in bacterial isolates grown under different conditions or niches [15], and is now a well-established method for examining differential gene expression (DE) in bacterial pathogens [16]. Here, we performed RNA-seq on strains from five of the CF cases that we have recently described [14] to catalogue both within-host and convergent transcriptional evolution during long-term *B. pseudomallei* infection in the CF lung. Paired isolates representing the initial and the most recent cultures available from each patient were compared. *B. pseudomallei* cultures were grown in an artificial sputum medium [17, 18] to mimic the conditions found in the CF lung environment.

## Methods

### CF isolates

The *B. pseudomallei* strains used in this study are summarised in Table 1. The history and genomic analysis of these cases and strains are detailed elsewhere [14].

**Table 1.** Summary of the genetic mutations and differentially expressed genes between paired, sequential *B. pseudomallei* isolates obtained from five CF patients (adapted from [14])

Patient	Initial and final isolate IDs	Time between collection (months)	No. of mutational events (Chr I, Chr II)	No. of genes affected by mutations (Chr I, Chr II)	No. of DE genes (Chr I, Chr II)	No. of DE genes (downregulated, upregulated)
CF6	MSHR5651, MSHR5654	27	24 (14, 10)	29 (14, 15)	229 (69, 160)	229 (124, 105∗)
CF8	MSHR8436, MSHR8437	46	12 (7, 5)	39 (5, 34)	32 (1, 31)	32 (32†, 0)
CF9	MSHR5662, MSHR5670	55	112	79 (40, 39)	792 (381, 411)	792 (558, 234)
CF10	MSHR8438, MSHR8440	10	0	0	0	0
CF11	MSHR8441, MSHR8442	14	15	38 (10, 28)	169 (62, 107)	169 (68‡, 101)

∗Seven of these genes are upregulated due to a 30× duplication affecting these loci in MSHR5654.

†Twenty-nine of these genes are downregulated due to a 35 kb deletion affecting these loci in MSHR8437.

‡Twenty-five of these genes appear as downregulated due to a 10× duplication affecting a 36.7 kb locus in MSHR8441 compared with only a 2× duplication in MSHR8442 [14].

### Artificial sputum medium

This medium was made as described elsewhere [17, 18], with modifications detailed here. Antibiotics were not used to maintain media sterility due to concerns that their addition would alter expression profiles. Due to the impracticality of its filtration [19], 1 g porcine stomach mucin (Sigma-Aldrich) dissolved in 40 ml ultrapure water was autoclaved prior to use. All other solutions were sterilised using a 0.22 µM vacuum filter, apart from the UV-irradiated egg yolk emulsion (Oxoid), which was treated aseptically. A stock solution of diethylenetriaminepentaacetic acid (Sigma-Aldrich) was made by dissolving 59.5 mg into 5 ml very basic water (pH=14). CaCl_2_ was added at a final concentration of 0.22 g l^−1^ (J. Manos, personal communication). Final concentrations of the components were: 10 g mucin l^−1^, 1.39 g salmon sperm DNA l^−1^ (Sigma-Aldrich), 5 g NaCl l^−1^, 2.2 g KCl l^−1^, 0.22 g CaCl_2_ l^−1^, 5 g casein acid hydrosylate l^−1^ (Sigma-Aldrich), 10 g BSA l^−1^ (Roche Diagnostics), 0.005 % diethylenetriaminepentaacetic acid and 0.5 % egg yolk emulsion. Each batch was tested for sterility prior to use by plating 100 µl onto Luria–Bertani (LB) agar (Oxoid) and incubating aerobically for 24 h. pH was tested using an aliquot of the medium to ensure it was within the desired range (pH ~6.5–7). The medium was stored at 4 °C for no longer than 4 weeks prior to use.

### Viability counts

Two sets of viability counts were performed for this study. The first was conducted to determine the number of c.f.u. at OD_590_, which enabled us to standardise the starting number of cells inoculated into the artificial sputum medium. The second was conducted to verify the final concentration of cells across all CF isolates, which enabled us to determine the number of cells for nucleic acid extraction to ensure that approximately equal cell amounts were processed for each pair. The CF isolates were subcultured from glycerol stocks onto LB agar at 37 °C for 24 h. Cells were suspended into PBS followed by spectrophotometric measurement at OD_590_ in a WPA CO 8000 cell density meter (Biochrom). Tenfold dilutions and plating of cultures onto LB agar were carried out, followed by enumeration at 24 h. Viable counts demonstrated that all CF isolates exhibited a similar cell density when normalised to an OD_590_=1.0 (range 1.3×10^8^ to 4.9×10^8^ cells). Based on these counts, the starting amount of culture for the CF isolates was standardised to 10^5^ c.f.u. for all subsequent experiments.

### Growth curves in artificial sputum medium

To minimize laboratory passage, each culture was again subcultured from the original glycerol stocks onto LB agar at 37 °C for 24 h, followed by a replication of OD_590_ measurements as determined previously. Based on the viability count data, samples were then diluted to 10^6^ c.f.u. ml^−1^ in PBS. One hundred microlitres of this suspension (~10^5^ c.f.u.) was used to inoculate 1.9 ml sputum medium, which was aliquoted into 14 ml Nunc round-bottom culture tubes (Thermo Fisher Scientific). Due to biosafety concerns, cells were grown in closed-capped tubes. The cultures were incubated at 37 °C by shaking in an orbital incubator shaker (model BL8500; Bioline) at 50, 200 or 230 r.p.m. for 44 h. Growth curves were obtained by measuring OD_590_ at regular intervals over this period using un-inoculated sputum medium as the control blank. Shaking at 50 r.p.m. was initially performed to mimic the low-oxygen conditions of the CF lung; however, this speed caused heavy sedimentation of cells and media components, and biofilm formation at the aerobic interface, both of which led to a decrease in optical density values over time and unpredictable, non-uniform growth. Similarly, shaking at 230 r.p.m. was too vigorous for the cells, as observed by inconsistent, non-reproducible viable counts. When shaking at 200 r.p.m., highly reproducible optical density values that correlated with viability counts were obtained (Fig. S1, available with the online version of this article), and the medium did not readily sediment. This speed was therefore used for subsequent experiments, including for RNA harvest. Viability counts were performed at the time of harvest to ensure uniformity of cell concentrations across all isolates.

### RNA extraction from isolates grown in artificial sputum medium

The CF strains were grown in duplicate lineages prior to being pooled for extraction, as detailed above. This step was carried out to minimise the chance of rare but significant laboratory-generated mutation(s) affecting gene expression in one of the lineages. In addition, two independent extractions were performed for all isolates, apart from the latter CF11 strain (MSHR8442), where three RNA extractions were carried out. Based on the growth curve analysis, nucleic acids for all cultures were extracted at late log phase (17 h). At the point of harvest, the OD_590_ of each replicate was measured to ensure that consistent cell density had been obtained prior to combining replicates; final viability counts were also performed. Due to the highly labile nature of bacterial mRNA, two 100 µl aliquots for each strain were immediately placed into 200 µl RNAprotect (Qiagen) and incubated for 5 min to preserve their transcription profiles. Cells were pelleted by centrifugation at 5000 ***g*** for 10 min, and the supernatant discarded. Total RNA was extracted using the RNeasy Protect bacteria mini kit (Qiagen). *B. pseudomallei* cells were lysed following the protocol for genomic DNA extraction [20], with an extended incubation time in proteinase K and lysozyme of 1.5 h. Lysates were loaded onto the RNeasy mini columns and extractions were carried out according to the manufacturer’s instructions, including the recommended on-column DNase I digestion. In our hands, we found this DNase I treatment to be insufficient for removing all contaminating DNA, so extractions for RNA-seq were further treated using a TURBO DNA-*free* kit (Ambion). For each sample, 35 µl extracted RNA was incubated with 6 U TURBO DNase at 37 °C for 32 min. The remaining RNA was not treated with this second round of DNase; instead, this sample was used as template for PCR contamination screening, as described below. All samples were transferred to clean RNase/DNase-free tubes for downstream processing.

### RNA quality control

To verify the removal of DNA from the total RNA extractions, two contamination screens were performed. The first was used to determine the removal of salmon sperm DNA, and the second was to determine the removal of *B. pseudomallei* DNA. Both the pre- and post-treated RNA samples were used to test for contamination, in duplicate, with the former acting as the positive control. The RNA samples were diluted 1/10 into molecular-grade H_2_O (Fisher Biotech) prior to PCR. Identification of residual salmon sperm DNA was investigated by targeting the mitochondrial 12S rDNA region of vertebrates [21]. Primers 12 S-6F (5′-CAAACTGGGATTAGATACC-3′) and B-12S-9R (5′-AGAACAGGCTCCTCTAG-3′) were used at a final concentration of 1 µM in a mix containing 1× PCR buffer (Qiagen), 1 U HotStarTaq, 0.2 mM dNTPs, 1 µl template and molecular-grade H_2_O in a 15 µl total reaction volume. Thermocycling conditions comprised 94 °C for 5 min, followed by 35 cycles of 94 °C for 30 s, 52 °C for 30 s and 72 °C for 30 s, and a final extension at 72 °C for 2 min. Amplicons were detected by agarose gel electrophoresis.

Real-time PCR was used to detect *B. pseudomallei* DNA contamination. The *mmsA* (methylmalonate-semialdehyde dehydrogenase) housekeeping gene was targeted using the primers Bp_266152_3012-F1-flap (5′-AATAAATCATAAACGTGAGGCCGGAGATGT-3′) and Bp_266152_ 3012-R1-flap (5′-AATAAATCATAAGACCGACATCACG CACAGC-3′) in combination with a *B. pseudomallei*-specific TaqMan MGB probe, 266152-T_Bp (5′-VIC-CGGTCTACACGCATGA-3′), as previously described [22], with the following modifications: 0.2 µM probe and 0.4 µM each primer was used, reactions were carried out in a 5 µl total reaction volume, and cycling was performed to 50 cycles.

### RNA storage, shipment and RNA-seq

For each sample, 20 µl total RNA was added to an RNAstable tube (Biomatrica), gently mixed with the preservation agent and left to air-dry in a biosafety cabinet for 48 h. Samples were shipped at ambient temperature to Macrogen (Geumcheon-gu, Seoul, Republic of Korea) for RNA-seq. Ribosomal RNA was removed by treatment with the Ribo-Zero rRNA removal kit for bacteria (Epicentre), followed by 100 bp paired-end, stranded library construction using the TruSeq rapid SBS Kit (Illumina). Libraries were sequenced on either the HiSeq2000 or the HiSeq2500 platform (Illumina). All samples were extracted from two separate experiments to account for biological variation, except for MSHR8442, which was extracted thrice. Between 36 and 80 million reads were generated for each sequence, corresponding to between 3.6 and 8.1 billion bp each.

### RNA-seq analysis

**Fig. 1.**
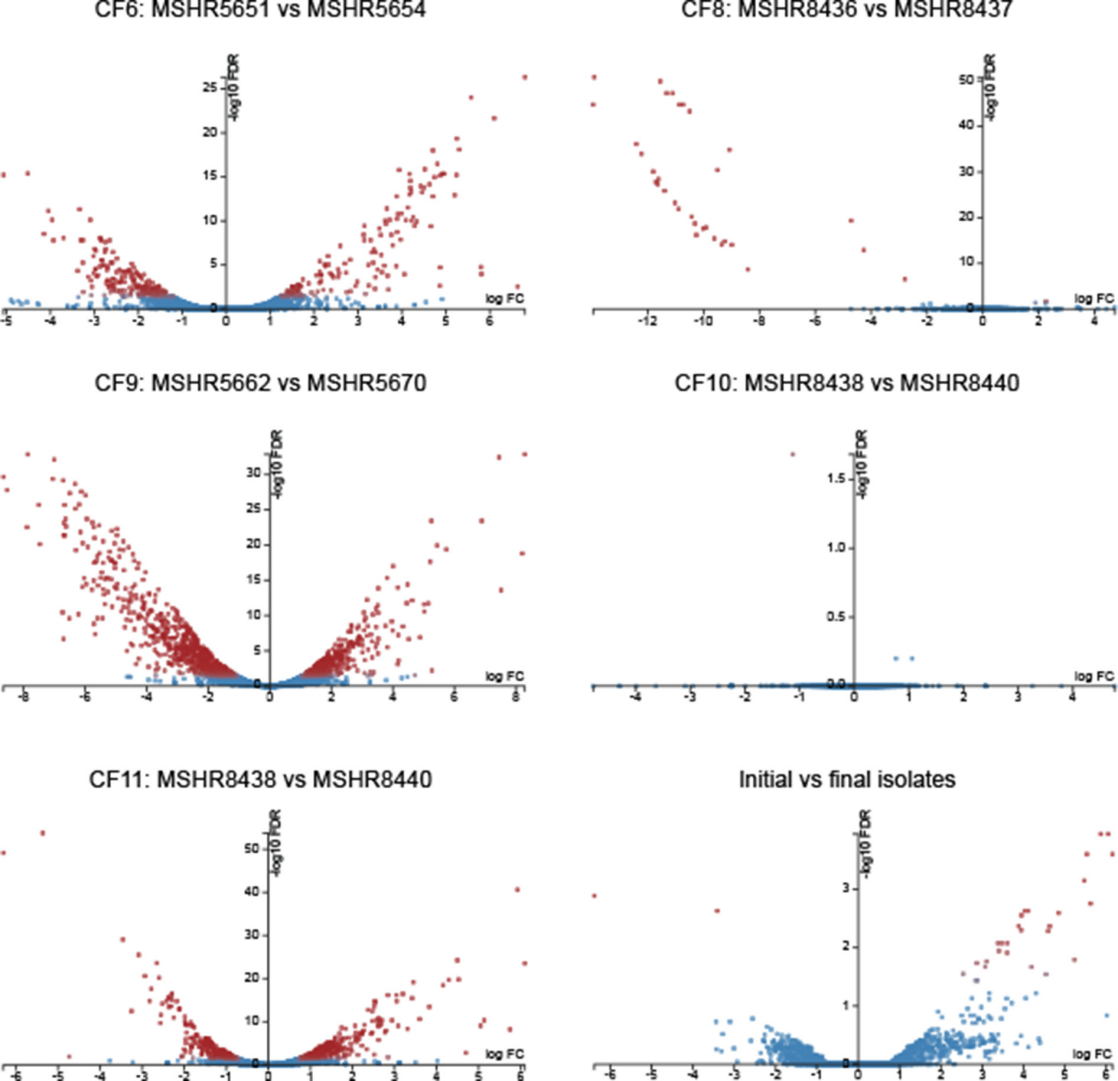
Degust volcano plots showing differentially expressed (DE) genes between paired *B. pseudomallei* isolates retrieved from five CF lungs, and between initial and latter isolates. Four of the five pairs exhibited DE; CF10, with the shortest time between isolates, exhibited no genetic or significant transcriptomic changes. CF6, CF8, CF9 and CF11 pairs were separated by 229, 32, 792 and 169 DE loci, respectively. The 32 DE loci in CF8 were all downregulated; DE genes in CF6, CF9 and CF11 were downregulated or upregulated. Nineteen loci were differentially expressed between initial and latter isolates, of which 17 were upregulated. Blue, genes with no significant DE; red, genes with significant DE. FDR, false discovery rate; FC, log_2_ fold change.

Illumina read filtering was first performed with Trimmomatic v0.33 using the following parameters: TruSeq2-PE adapter removal, leading=3, trailing=3, sliding window=4 : 15 and minimum length=36. Reads were mapped to the prototypic *B. pseudomallei* K96243 reference genome (RefSeq IDs NC_006350 and NC_006351 for chromosomes 1 and 2, respectively [23]) using Bowtie 2 v2.2.1 [24]. For within-patient analyses, the initial patient genomes were downloaded from the National Center for Biotechnology Information (GenBank references JYBG00000000, JYBH00000000, JYBI00000000, JYBJ00000000 and JYBK00000000 for MSHR5651 [CF6], MSHR8436 [CF8], MSHR5662 [CF9], MSHR8438 [CF10] and MSHR8441 [CF11], respectively) and annotated using Prokka v1.12 [25] with the K96243 proteins as priority (using the ‘−−proteins’ flag) prior to Bowtie 2 v2.3.4 analysis. Transcript quantification was performed with HTSeq (v0.6.1p1) [26] using the intersection non-empty mode and −−stranded=reverse parameters. DE analysis was carried out using the glmFit function of edgeR v3.18.1 [27], implemented in the online Degust 3.1.0 tool (http://degust.erc.monash.edu/). DE loci were visualised using the ‘volcano plot’ function within Degust. Several different groups were compared to determine DE. The first analyses compared initial and latter isolates within CF patients (Table 1) without summing technical replicates (i.e. the RNA-seq data from each independent experiment of a single strain) to identify DE within each patient. To determine convergent DE loci, we summed the reads for each technical replicate prior to analysis and then compared all initial versus all latter isolates, with the latter CF10 isolate excluded due to a lack of DE in this strain and the initial CF11 isolate excluded due to >3 years of infection prior to its isolation [14]. For all analyses, significant DE was defined as a log_2_ fold change of ≥1.5 and a false discovery rate of ≤0.01. To improve visualisation of DE loci in the volcano plot of the initial and latter comparison (Fig. 1), highly expressed DE loci in only a single strain were omitted.

## Results

### DE among CF isogenic pairs

DE was observed in four of the five CF pairs, with only CF10 failing to yield significant transcriptional differences (Fig. 1). We have previously shown that no genetic variants separate the CF10 strains, which had the shortest time between collection of only 10 months [14]. This lack of significant transcriptional differences rules out epigenetic effects (e.g. DNA methylation) on gene expression between the CF10 isolates, at least under the tested growth conditions, and illustrates that RNA-seq is a robust methodology that is not readily prone to false-positive results.

Of the four pairs with significant DE, CF8 had the fewest with 32 loci, followed by CF11, CF6 and CF9 with 169, 229 and 792 DE loci, respectively (Fig. 1, Table 1). These paired isolates were collected 46, 14, 27 and 55 months apart, respectively. There was good correlation between the proportion of DE loci and the genome-wide mutations catalogued between these pairs [14], with 12, 15, 24 and 112 mutational events (i.e. SNPs, indels, deletions or gene duplications) identified in CF8, CF11, CF6 and CF9, respectively (Table 1). The elevated number of mutations seen in CF9 is due to a *mutS* mutation in the latter strain, which confers a hypermutator phenotype, the first time hypermutation has been described in *B. pseudomallei* [14]; this in turn contributes to a high number of DE genes. However, when comparing the ratio of DE genes to mutational events, CF11 had the highest proportion of DE genes (11.3), followed by CF6 (9.5), CF9 (7.1) and CF8 (2.7). There was a significant skew towards DE genes located on chromosome II, which contains a lower proportion of housekeeping genes than chromosome I [23]. Despite encoding only 44 % of the genome by size and 41 % of coding sequences, chromosome II loci were significantly overrepresented in the non-hypermutator CF pairs (Pearson’s χ^2^ test *P*<0.001), with between 63 and 97 % of the DE genes residing on chromosome II. In CF9, there was a non-significant trend towards chromosome II loci, with 52 % of DE loci located on this chromosome, pointing to the more random nature of mutations in the hypermutator compared with the other cases (Table S1). In addition, to detect any significant DE in the pangenome, we performed DE analysis using the initial strain from each patient as a reference and compared these results with those obtained using K92643 (Tables S1 and S2). On balance, the within-patient analyses were almost identical to those using the K96243 reference, with most discrepancies attributable to differences in gene annotation methods.

### Many DE genes are absent in the strain found in chronic-carriage melioidosis patient 314 (P314)

We compared the DE loci in the CF strains to those genes mutated in the strain found in chronic-carriage melioidosis case P314. P314 has the longest *B. pseudomallei* infection ever documented, and despite multiple eradication attempts, continues to harbour this bacterium in the airways since first being diagnosed in 2000. We have previously shown that the genome of a 139 month isolate from P314, MSHR6686, shows dramatic adaptation to the lung environment, including the loss of 285 kb of chromosome II at four separate locations that collectively encompass 221 genes [28]. When compared with genes lost in MSHR6686, there was a 9, 15, 20 and 97 % overlap in the latter isolates from CF9, CF6, CF11 and CF8, respectively (Table S1). The proportion of downregulated genes varied across this dataset, ranging from 13 % for CF11 to 100 % for CF8, demonstrating that the effect on gene expression at these loci is not unidirectional, with certain overlap loci in fact being upregulated in CF6, CF9 and CF11. Of note, all 29 genes (*BPSS1131–BPSS1159*) that were lost due to a large deletion in the latter CF8 isolate [14] were also absent in MSHR6686 [28], providing further evidence of their dispensability for long-term *B. pseudomallei* survival in the mammalian host. As expected, DE showed dramatic downregulation of between 8 and 14 log_2_ fold (341 to 15 886×) of these loci (Table S1).

### DE of surface antigens in the CF6, CF9 and CF11 pairs

*B. pseudomallei* produces four capsular polysaccharides (CPS-I to -IV) and a lipopolysaccharide (LPS). However, only CPS-I (encoded by *BPSL2786–BPSL2810*) and LPS (encoded by *BPSL1936* and *BPSL2672–BPSL2688*) are associated with virulence in mammals [29, 30]. Our previous genomic analysis of the CF pairs identified missense mutations affecting the LPS loci *wzt* (*BPSL2681*) and *rmlA* (*BPSL2685*) in CF6, a missense mutation affecting a putative LPS biosynthesis gene *BPSL1119* in CF11 and a CPS-I frameshift mutation affecting *wcbA* (*BPSL2809*) in CF11 [14].

Consistent with being under heavy selection during chronic infection, we observed DE of several surface polysaccharide loci in the CF6, CF9 and CF11 pairs, the most dramatic of these being in CF9, with 46 downregulated surface polysaccharide genes (Table S1). The LPS loci *wbiE* (*BPSL2676*) and *wbiD* (*BPSL2677*) were downregulated by ~1.8 fold (3×) in the latter CF9 isolate. In addition, the poorly characterised LPS biosynthesis-related membrane protein loci *BPSS1683*–*BPSS1685* were downregulated by ~5.9 fold (60×). This isolate also exhibited downregulation of all CPS-I loci (except *wcbC*) of between 1.7- and 3.1-fold (3 to 8×). In contrast, the latter isolate from CF11 showed upregulation of the CPS-I loci *BPSL2793*–*BPSL2797* (*wcbN-wcbM-gmhA-wcbL-wcbK*) when compared with its initial isolate, with increases ranging from 2.7 to 6.1-fold (7 to 69×). However, when compared with initial isolates from CF6, CF8, CF9 and CF10, expression of *BPSL2793–BPSL2797* in the latter CF11 strain was in fact downregulated (3.4 to 4.5-fold; 11 to 23×). This observation was confirmed as significant downregulation of these loci in the initial CF11 strain (by between 6.1- and 10.1-fold; 69 to 1098×) when compared with all other initial strains, rather than significant upregulation of these CPS-I loci in the latter CF11 strain.

Unlike CPS-I, expression of the CPS-II cluster (*BPSS0417*–*BPSS0429*) is induced when grown in water, suggesting that this polysaccharide plays a role in environmental survival [29]. One locus involved in CPS-II biosynthesis, *BPSS0425*, was downregulated (1.8-fold; 4×) in CF6, and the entire cluster was downregulated in CF9 (range 2.5- to 5.2-fold; 6 to 37×). Conversely, *BPSS0417* and *BPSS0418* were upregulated in CF11 (~1.6-fold; 3×). However, as with CPS-I, both CF11 strains exhibited significant downregulation of *BPSS0417* and *BPSS0418* when compared with initial strains from CF6, CF8, CF9 and CF10 (2.0- and 3.1-fold; 4 to 9×). The genes encoding CPS-III (*BPSS1825–BPSS1835*) were significantly downregulated in the latter isolates from CF6 (2.4- to 3.1-fold; 5 to 9×) and CF9 (5.6- to 7.5-fold; 50 to 178×). Finally, two genes within the CPS-IV cluster (*BPSL2769–BPSL2785*) were downregulated in CF9 (*BPSL2782* and *BPSL2785*; 1.8- to 2.8-fold; 3 to 7×), but 11 of 17 loci from this cluster were upregulated in CF11 by 1.7- to 5.9-fold (*BPSL2769*, *BPSL2775–BPSL2784*). However, unlike CPS-I and CPS-II, this pattern of upregulation in both CF11 isolates was maintained for CPS-IV loci *BPSL2769* and *BPSL2775*–*BPSL2781* even when compared with the initial CF6, CF8, CF9 and CF10 isolates (2.0- to 4.0-fold; 4 to 16×).

### DE of other virulence-associated loci

In addition to CPS-I and LPS, *B. pseudomallei* encodes for several other virulence factors that enhance organism survival and replication upon infection or that subvert or disarm host defences. These factors include adhesins, flagella, fimbriae, pili, specialised secretion systems, actin motility proteins, secreted factors and secondary metabolites [30]. Although virulence factors are often critically important during the acute stages of infection, they can become disadvantageous for long-term survival, presumably due their immunogenicity [12, 28]. Consistent with loss-of-virulence as a pathoadaptive mechanism in chronic *B. pseudomallei* infections, we have previously documented missense mutations affecting the type 3 secretion system 3 (T3SS-3) gene *bsaW* in CF6, and *Burkholderia* biofilm factor A-encoding gene, *bbfA,* and fimbrial protein-encoding *BPSL1628* in CF9 [14]. When examining RNA-seq profiles, other virulence genes lacking genetic mutations were found to be significantly downregulated in the latter CF isolates. These loci included three type IV pilus 7 (TFP7) loci (*pilR*, *pilG* and *pilN*; 1.7-fold; 3×), the lysozyme inhibitor-encoding *BPSL1057* (3.4-fold; 11×), *Burkholderia* lethal factor 1 (3.2-fold; 10×), four T3SS-3 loci (*bsaS*, *bsaP*, *bsaO* and *bsaN*), and 15 flagellum loci in CF9 (mean 2.8-fold; 8×), and a trimeric autotransporter adhesin (*bpaC; BPSL1631*) in CF11 (1.6-fold; 3×). Of these, the four T3SS-3 loci are also missing in chronic P314 isolates. It is notable that no genetic mutations were identified in these virulence factor loci in the latter CF isolates, highlighting the advantage of using transcriptomics to detect patterns of within-host pathogen adaptation that are not observable with genomic data alone.

### High-level trimethoprim/sulfamethoxazole (SXT) resistance in *B. pseudomallei* involves *bpeEF*–*oprC* upregulation

The combination antibiotic SXT, the drug of choice in the eradication phase of melioidosis treatment [31], was administered to patients CF6, CF9 and CF11 during their *B. pseudomallei* eradication attempts (Table 2). Acquired resistance towards SXT emerged in the latter isolates from CF6 and CF11, and in midpoint isolates from CF9 [14]. The resistance-nodulation-division (RND) efflux pump, BpeEF–OprC, is responsible for widespread trimethoprim resistance in *B. pseudomallei* and has been implicated in SXT resistance [32]. BpeEF–OprC (*BPSS0292*–*BPSS0294*) expression is under the control of two LysR-type regulators, BpeT (*BPSS0290*) and BpeS (*BPSL0731*). We, therefore, expected to observe upregulation of *bpeEF*–*oprC* in CF strains with elevated SXT minimum inhibitory concentrations (MICs), consistent with defective *bpeT* or *bpeS* loci.

**Table 2.** Summary of clinical aspects and mechanisms of antibiotic resistance in *B. pseudomallei* isolates retrieved from five CF patients Antibiotics highlighted in bold were targeted towards *B. pseudomallei* eradication. Mutations in red are putative and remain to be functionally characterised. AMC, amoxicillin/clavulanate; AMR, antimicrobial resistance; DOX, doxycycline; F, female; IV Ab, intravenous antibiotic; LT, lung transplant; M, male; MEM, meropenem; na, not applicable; nd, not determined; TET, tetracycline; TN, tobramycin.

**Patient ID**	**Age at diagnosis (years) [sex]**	**No. of months between isolates**	**Treatment**	**AMR MIC (µg ml^−1^)**	AMR genetic mechanism(s)*,†	**DE loci confirming AMR mechanism(s)**	**Detected co-pathogens‡**	**Patient outcome**
CF6	21 [M]	27	**AMC, CAZ**, CIP, **MEM**, **SXT**	CAZ: ≥256	PenA^C69Y^+~30× *penA* duplication	↑22× *penA*	*P. aeruginosa*, *S. aureus*, *B. cepacia* complex	Died (~23 years old)
				CIP: ≥32	GyrA^Y77S^	na		
				SXT: ≥32	Ptr1^R20fs^+BpeT^T314fs^	↑38-111x *bpeEF*–*oprC*		
CF8	14 [M]	46	Nil IV Ab	MEM: 3–4; both isolates	Unknown	Unknown	*P. aeruginosa*, *S. aureus*	Unknown
CF9^§^	21 [M]	55	**CAZ**, **MEM**, **SXT**, TET; LT	DOX: 48	AmrR^L132P^+BPSL3085^A88fs^	nd	*P. aeruginosa*	Stable despite non-eradication
				MEM: 4	AmrR^L132P^	nd		
				SXT: ≥32	MetF^N162P^+Dut^N99S^+AmrR^L132P^	nd		
CF10	25 [F]	10	**CAZ**, **MEM**, TN	None detected	na	na	*P. aeruginosa*, *S. aureus*, *B. cenocepacia*	*B. pseudomallei* eradication; died (~34 years old)
CF11	10 [F]	14	**CAZ**, **AMC**, TN, **MEM**, **SXT**, **DOX**	SXT: ≥32; both isolates	Ptr1^R20-A22ins^+BpeS^V40I^ and ^R247L^	↑40-121× *bpeEF*–*oprC*	*P. aeruginosa*, *S. aureus*	Died (16 years old)
				CAZ: 12	~10× *penA* duplication (initial strain); ~2× *penA* duplication (latter strain)	↓4× *penA* in latter strain		
				DOX: 4–8; both isolates	AmrR^ΔV62-H223^+BPSL3085^S130L^	↑6-9× *amrAB*–*oprA*		
				MEM: 3	AmrR^ΔV62-H223^	↑6-9× *amrAB*–*oprA*		

*Resistance profiles and mechanisms shown for the latter isolate, unless otherwise specified.

†As reported by Viberg *et al*. [14].

‡From Geake *et al.* [13].

§The final isolate from CF9, MSHR5670, was not resistant to any clinically-relevant antibiotics; however, midpoint isolate MSHR5667 (collected 47 months after the initial isolate) was DOX resistant (48 µg ml^−1^), and all midpoint isolates (MSHR5665, MSHR5666, MSHR5667 and MSHR5669) were SXT resistant (≥32 µg ml^−1^).

The latter isolate from CF6, which encodes a T314fs mutation in *bpeT* and is highly resistant towards SXT (MIC≥32 µg ml^−1^), showed 5.2- to 6.8-fold upregulation of *bpeEF*–*oprC* (38 to 111×; Table 2). This isolate also harbours an R20fs mutation in *ptr1* (*BPSS0039*; *folM*), which encodes a pteridine reductase that is involved in SXT resistance [32]; this frameshift truncates Ptr1 from 267 to 91 residues. Both strains isolated from CF11 are also highly resistant to SXT (MIC≥32 µg ml^−1^) and encode the BpeS missense variants V40I and R247L (K96243 annotation) when compared with wild-type *B. pseudomallei* strains [14]. They also encode a three-residue in-frame insertion of R20-A22 in Ptr1 [14, 32]. Because both CF11 strains are SXT resistant, DE was determined by comparison with SXT-sensitive isolates in our dataset. Using this approach, significant upregulation was observed for BpeEF–OprC (5.3- to 6.9-fold; 40 to 121×; Table 2). DE was not observed for *ptr1* or other genes involved in the folate biosynthesis pathway in either the CF6 or CF11 isolates (Table S1).

### Ceftazidime (CAZ) resistance can occur by upregulation of *penA*

CAZ is a third-generation cephalosporin antibiotic that is the most commonly recommended therapy for the primary phase of melioidosis treatment [33]. In addition to SXT, the latter isolate from CF6 is highly resistant to CAZ (MIC≥256 µg ml^−1^) [14]. High-level CAZ resistance is often conferred by a C94Y substitution (C69Y using Ambler’s [34] numbering scheme) in PenA β-lactamase [35–37]. We have recently shown that the latter CF6 strain also harbours a ~30× duplication of a 7.5 kb region that encompasses *penA*; all 30 copies encode the C69Y variant of this enzyme [14]. Consistent with this duplication event, *penA* (*BPSS0946*) expression increased by 4.5-fold (22×) in the latter CF6 strain (Table 2). Six proximal genes (*BPSS0945*; *BPSS0948*–*BPSS0952*) were also upregulated by 3.1- to 4.7-fold (9 to 26×; Table S1). One of these, *BPSS0945*, is a peptidase and a putative virulence factor that may play a role in multinucleated giant cell formation [38].

A gene duplication event encompassing *penA* has also been documented in the CF11 isolates. The initial strain showed an elevated MIC towards CAZ (12 µg ml^−1^), corresponding with a ~10× duplication of a 36.7 kb region that includes *penA*, whereas the latter strain had a 2× duplication of this region and a wild-type CAZ MIC (2 µg ml^−1^) [14]. As expected, *penA* was downregulated by 2.1-fold (4×) in the latter isolate due to five times fewer copies of this gene (Table 2). Downregulation of other genes within the 36.7 kb locus ranged from 1.4- to 3.3-fold (3 to 10×; Table S1).

### Increased doxycycline MICs in CF11 are due to *amrAB*–*oprA* upregulation and *BPSL3085* mutation

Doxycycline was administered to CF11 in combination with SXT as part of a final attempt to eradicate *B. pseudomallei* [13, 33]. This lengthy administration led to a doxycycline MIC of 4–8 µg ml^−1^ in the CF11 isolates, both of which were retrieved post-treatment [14].

The RND efflux pump, AmrAB–OprA (*BPSL1802*–*BPSL1804*), efficiently effluxes aminoglycoside- and macrolide-class antibiotics [39]. We have recently shown that synergistic mutations affecting both its regulator AmrR (*BPSL1805*) and an *S*-adenosyl-l-methionine (SAM)-dependent methyltransferase (*BPSL3085*) led to doxycycline resistance in an Australian melioidosis case [40]. Consistent with this earlier work, both CF11 strains encode a large deletion in *amrR* (AmrR^ΔV62-H223^). This mutation results in a 2.6- to 3.2-fold (6 to 9×) upregulation of *amrAB–oprA* in these isolates (Table 2). In addition, a previously undocumented S130L mutation in *BPSL3085* was observed in both strains.

### Decreased meropenem susceptibility is generally conferred by *amrAB–oprA* upregulation

The latter isolate from CF11, a midpoint isolate from CF9 (MSHR5667), and both CF8 isolates possess elevated MICs towards meropenem (3–4 µg ml^−1^) [14]. Mutations in RND efflux pump regulators, especially AmrR, are responsible for this decreased susceptibility, and importantly, such cases are associated with more refractory treatment and poorer patient outcomes [41]. Alongside its elevated doxycycline MIC, the AmrR^ΔV62-H223^ mutation in the latter CF11 isolate also confers decreased meropenem susceptibility. The midpoint CF9 isolate, MSHR5667, was not examined using RNA-seq or quantitative PCR in our study, but we hypothesise that its AmrR^L132P^ mutation causes ~2–4-fold upregulation of *amrAB*–oprA in a similar fashion to the CF11 isolates. Curiously, the CF8 isolates had no observed mutations or DE loci involving efflux pumps when compared with wild-type strains, and the basis for the elevated MIC in these isolates remains elusive. Growing these isolates in the presence of low-level meropenem to induce expression prior to RNA-seq/quantitative PCR [42] may be required to identify the transcriptional mechanism/s underpinning this phenotype.

### Evidence of convergent DE between early and latter CF isolates

Finally, we performed a comparison of expression profiles from all CF cases to identify a signal of convergent gene expression (pathoadaptation) across early versus latter isolates. To yield the most robust and relevant analysis, we excluded the latter isolate from CF10 due to a lack of DE in this strain, and the initial isolate from CF11, which was retrieved >3 years after infection and had already undergone substantial genetic and transcriptional modifications. Exclusion of both strains was supported by a lack of convergent signal when they were included in the analysis (data not shown). Using these parameters, 17 genes were found to be significantly upregulated, and 2 were significantly downregulated (Table S3). Five (26 %) loci encode for hypothetical proteins with no known function, of which four were upregulated. Among the convergently upregulated genes with known function was the RND efflux pump BpeEF–OprC (4.8- to 6.1-fold; 28 to 69×), the CydAB cytochrome *bd* quinol oxidase (5.5- to 5.9-fold; 45 to 60×), and the quorum sensing *hhqABCDEFG* (*BPSS0481*–*BPSS0487*) operon (3.4- to 4.1-fold; 11 to 17×). The downregulated locus, *BPSS0351*, encodes the MerR family transcriptional regulator CueR (3.4-fold; 11×).

## Discussion

The melioidosis pathogen, *B. pseudomallei*, is an uncommon pathogen in CF, with fewer than 30 cases documented worldwide to date. However, it is an important pathogen in CF airways due to its ability to persist despite treatment and its association with accelerating respiratory decline [13]. We have recently performed comparative genomic analysis of isogenic strains collected between 4 and 55 months apart from the airways of the patients in seven of these cases [14]. Here, we sought to further characterise these chronic cases by examining the transcriptomes of five paired *B. pseudomallei* isolates retrieved between 10 and 55 months apart. Although our sample numbers are modest, our study represents more than one-sixth of globally reported melioidosis cases in CF to date.

Isolates were cultured in an artificial CF sputum medium [18] to mimic their original *in vivo* environment. Under these conditions, DE was detected in four of the five cases and ranged from 32 to 792 genes, with the hypermutator strain from CF9 contributing the greatest number of DE loci (Table S1). Interestingly, when compared with the number of genetic changes occurring in each isolate pair, the latter isolate from CF11 had a higher proportion of DE loci to mutations (11.3) than CF9 (7.1), demonstrating that hypermutation does not necessarily lead to a similarly high number of transcriptional differences. The one case with no DE, CF10, exhibited no genetic changes (i.e. SNPs, small indels, copy-number variants or large deletions) and had the shortest time between isolate collection at 10 months [14]. All other cases encoded genetic differences between pairs. The DE genes fell into several functional categories (Table S1), reflecting the diversity and versatility of pathoadaptive pathways in *B. pseudomallei*. Our RNA-seq analysis revealed that many of the DE genes were absent in the chronic P314 strain (range 9–97 %), providing further evidence that these loci are not required for long-term survival in the airways. Perhaps most striking was the observation that nearly one-third (32 %) of DE genes lack a known function, highlighting the relative paucity of functional studies into this important yet under-recognised pathogen.

*B. pseudomallei* has a ~7.3 Mbp genome that is encoded on two replicons; a ~4.1 Mbp ‘housekeeping’ chromosome I and a ~3.2 Mbp ‘accessory’ chromosome II. The genome of archetypal strain K96243 consists of 3460 and 2395 coding sequences on chromosomes I and II, respectively [23]. There was a greater proportion of DE genes (between 52 and 97 %) on chromosome II in all cases, despite its smaller size and fewer coding sequences. We have previously shown that a greater proportion of mutational events affect chromosome II of *B. pseudomallei* in a long-term chronic-carriage isolate from P314 [28]. The skew towards DE loci on chromosome II in chronic *B. pseudomallei* isolates points towards a lesser role for chromosome II loci in bacterial survival and persistence within the human host, which is reflected by the greater degree of reductive evolution affecting this replicon [14, 28]. In their study of the transcriptional landscape of *B. pseudomallei*, Ooi and co-workers found that only ~28 % of chromosome II genes were expressed under a single condition, compared with ~72 % of chromosome I genes [43]. Taken together, these results support the accessory role of the chromosome II replicon in *B. pseudomallei*.

Attenuation of immunogenic surface antigens and other virulence factors are hallmarks of chronically persistent infections across many pathogenic bacterial species, including *B. pseudomallei* [14, 28]. Encoded by the 34.5 kb *wcb* operon *BPSL2786*–*BPSL2810* [44, 45], the *B. pseudomallei* CPS-I is a potent virulence determinant that imparts high-level serum resistance and facilitates phagocytic evasion [45]. This capsule is also intact in the equine-adapted *B. pseudomallei* clone, *Burkholderia mallei*, where it and has been shown to be essential for its virulence [46, 47]. Our prior genomic analysis identified only a single CF pair with mutated CPS-I in CF11. This frameshift mutation in *wcbA* results in a truncated protein [14] that likely causes reduced, although not abolished, CPS-I production [48]. The DE analysis provides further evidence of CPS-I inactivation in the CF pairs, with downregulation of all but one of the CPS-I loci in the latter CF9 isolate, and downregulation of *wcbN-wcbM-gmhA-wcbL-wcbK* in both CF11 isolates. Although CPS-III is not required for virulence, it is noteworthy that this locus was also downregulated in CF9 and CF11, as it has been previously shown that CPS-III expression is tied to that of CPS-I genes [43].

Like CPS-I, the *B. pseudomallei* LPS is required for capsule biosynthesis, virulence and serum resistance [49]. Its immunogenic outer-membrane component is readily recognised by the host innate immune system [50], which makes LPS a target for inactivation in chronic bacterial infections. We have previously uncovered missense mutations in the LPS *wzt* and *rmlA* loci of CF6, and a missense mutation affecting *BPSL1119* in CF11 [14]. DE analysis identified additional evidence for reduced or abolished LPS production in a third CF case, CF9, due to the significant downregulation of *wbiD*, *wbiE* and *BPSS1683*–*BPSS1685*. Prior work has suggested that CPS-I and LPS may be disadvantageous for *B. pseudomallei* persistence due to their virulence potential and immunogenicity [28]. By examining both genomic and transcriptomic modifications over time, it is now clear that these capsule clusters, in their wild-type form, pose a major issue for successful long-term *B. pseudomallei* persistence in the CF lung, with the bacterium either mutating or downregulating key genes in CPS-I and LPS pathways. We also observed genetic mutation or transcriptional downregulation of other virulence genes in latter CF strains including TFP7 loci, *Burkholderia* lethal factor 1, T3SS-3 loci and flagellum loci, suggesting that these loci are similarly detrimental to long-term *B. pseudomallei* survival in the human host. Importantly, the RNA-seq data identified additional cases of surface antigen and virulence factor abrogation that were not observable with only genomic data. This finding underscores the importance of using both genomic and transcriptomic approaches to identify the functional consequences of within-host evolution of chronic bacterial infections.

SXT is used during the eradication phase of melioidosis treatment and is recommended for post-exposure prophylaxis [51]. We have previously shown that the latter isolate from CF6, and both isolates from CF11, had developed high-level (≥32 µg ml^−1^) SXT resistance over the course of treatment. These elevated MICs were proposed to be due to mutations within BpeEF–OprC efflux pump regulators (BpeT T314fs in CF6, and BpeS V40I and R247L in CF11) alongside mutations affecting the R20 residue of Ptr1/FolM (R20fs in CF6; R20-A22 duplication in CF11) [14]. Here, we have demonstrated that the efflux pump regulatory mutations cause a dramatic upregulation of *bpeEF*–*oprC* in these strains of between 5.2- and 6.9-fold (38 to 121×), mirroring expression levels in *bpeS* and *bpeT* laboratory-generated mutants with high-level SXT resistance [32]. Our results confirm those of Podnecky and colleagues [32] showing that upregulation of *bpeEF*–*oprC* via BpeS or BpeT dysregulation, together with Ptr1/FolM alteration, leads to a significant increase in SXT MICs that would render this antibiotic ineffective *in vivo*. RNA-seq is, thus, a useful tool for observing the functional consequences of regulatory mutations that control RND efflux pump expression.

In addition to SXT resistance, the initial isolate from CF11 (12 µg ml^−1^) and the latter isolate from CF6 (≥256 µg ml^−1^) are resistant to CAZ. Our prior genomic study showed that CAZ resistance in the initial CF11 strain was due to a 10× duplication of a 36.7 kb region encompassing the β-lactamase gene, *penA*, the first time that gene duplication has been shown to confer CAZ resistance in *B. pseudomallei*. In contrast, a 2× duplication of this region in the latter strain did not raise the CAZ MIC above wild-type levels [14]. Similarly, the latter strain from CF6 exhibits a 30× duplication of a 7.5 kb region encompassing *penA*; however, all 30 copies encode a C69Y missense mutation, which by itself causes high-level (≥256 µg ml^−1^) CAZ MICs [35]. RNA-seq confirmed the *penA* duplications, with the 30× variant from CF6 observed as 4.5-fold (22×) upregulation. Similarly, a 2.1-fold (4×) downregulation of *penA* in the latter strain from CF11 was linked to a 5× greater copy number in the early strain [14]. Thus, RNA-seq provides excellent correlation with gene copy number variation determined from whole-genome sequence coverage data. Taken together, the combined genetic and transcriptional changes affecting antibiotic resistance genes in the CF airways-adapted *B. pseudomallei* strains illustrates both the intractability of eradicating chronic bacterial infections and the unintended consequences of prolonged antibiotic use in CF treatment.

Although determining within-host transcriptional differences in longitudinal isolates yields valuable insights into the infection dynamics within individual patients, identifying convergent transcriptional changes provides a potential means to predict pathogen behaviour and evolution across multiple CF cases in a relatively straightforward manner. Such predictability could conceivably be exploited to improve the diagnosis or treatment of intractable CF infections, or ideally, to prevent them from progressing in the first place. Therefore, a major objective of this study was to identify evidence of convergence in *B. pseudomallei* gene expression during its transition to a chronic infection. Despite the small number of CF melioidosis patients available for this study, a signal of convergent pathoadaptation was identified between the initial and latter isolates, with 19 significantly DE loci identified, 17 of which were upregulated (Table S3). This convergence is noteworthy given the large size of the *B. pseudomallei* genome and the many redundant pathways that could lead to similar adaptive phenotypes, a phenomenon that is well-recognised in *P. aeruginosa* [52]. One advantage of identifying convergence using transcriptomics rather than genomic data is that it can reveal the transcriptional consequence of multiple genetic mutations; for example, we have observed that multiple missense mutations in the RND efflux pump regulator AmrR lead to the same transcriptional outcome of *amrAB*–*oprA* upregulation [41]. As such, RNA-seq data can simplify the identification of convergently expressed loci that can be dysregulated by multiple genetic variants.

The development of antibiotic resistance is a recurring theme in *P. aeruginosa* isolated from the CF airways [12], and we have recently shown that the same adaptive phenomenon can be observed in the genome of *B. pseudomallei* in response to prolonged, high-dose antibiotic therapy [14]. It was, therefore, not surprising to identify the convergent upregulation of *bpeEF*–*oprC* (4.8- to 6.1-fold; 28 to 69×), which was significantly upregulated in two of the four patients with DE (CF6 and CF11), and which led to SXT resistance as discussed above. The second convergently upregulated locus was the *cydAB* operon (*BPSL0501* and *BPSL0502*), which encodes cytochrome *bd* quinol oxidase (5.5- to 5.9-fold; 45 to 60×); this locus was significantly differentially expressed in CF6 and CF9. CydAB is an aerobic terminal oxidase that oxidizes ubiquinol-8 and reduces oxygen to water under oxygen-limiting conditions. This enzyme is better able to scavenge oxygen under microaerobic conditions compared with cytochrome *o* oxidase, which otherwise predominates as the terminal respiratory enzyme in electron transport-associated energy production [53]. Voggu and colleagues demonstrated that non-pathogenic *Staphylococcus* species were better able to resist *P. aeruginosa* antagonism compared with *Staphylococcus aureus* due to the insensitivity of the non-pathogenic staphylococci cytochrome *bd* quinol oxidases to the presence of the small respiratory inhibitors hydrogen cyanide and pyocyanin, which are commonly secreted by *P. aeruginosa* in the CF lung [54]. Thus, it is feasible that the convergent upregulation of *cydAB* loci represents a defence mechanism employed by *B. pseudomallei* to counteract the toxic effects of small respiratory inhibitors produced by *P. aeruginosa* in the CF lung. In support of this hypothesis, *P. aeruginosa* was co-isolated in all five CF cases examined in this study [14]. Alternatively, *cydAB* upregulation may simply represent a physiological response to the oxygen-limited environment of the CF airways, as its expression is known to be induced in *B. pseudomallei* in hypoxic conditions [55]. Under hypoxia, many pathogens including *B. pseudomallei* become less susceptible to conventional antibiotics, which are typically effective under aerobic conditions, but more susceptible to antibiotics that target anaerobic infections. One example is the nitroimidazole class of antibiotics [55], which reduce their nitro group only under hypoxic conditions, causing DNA strand breakage and bacterial cell death [56]. This phenomenon may explain the difficulties with chronic *B. pseudomallei* eradication using conventional antibiotics like CAZ and SXT, and raises the exciting but not-yet-tested possibility that nitroimidazoles may be a highly effective therapeutic option for chronic, hypoxia-adapted *B. pseudomallei* infections such as those adapted to the CF airways.

A third convergently upregulated locus, the quorum sensing operon *hhqABCDEFG* (3.4- to 4.1-fold; 11× to 17×), is homologous to the *B. cepacia* complex *hmqABCDEFG* operon [57]. This operon synthesises a class of compounds known as 4-hydroxy-3-methyl-2-alkylquinolines (HMAQs), the methylated counterparts of 2-alkyl-4(1H)-quinolones (AHQs; also known as HAQs). AHQs were first recognised in *P. aeruginosa* and are produced by the signalling system *pqsABCDE* [58]. This cluster produces over 50 different AHQs, and these compounds exhibit diverse biological activities that enable cell-to-cell communication within and between bacterial species and the regulation of various functions including secondary metabolism, virulence, antibacterial activity and biofilm formation [58]. In contrast, little is currently known about the role of HMAQs and AHQs in *Burkholderia* spp. [57]. The AHQ precursor molecule 2-heptyl-4(1H)-quinolone (HHQ) that is produced by *P. aeruginosa* actively suppresses the host innate immune response [59], a role that could be shared by *B. pseudomallei* HHQ. A second possibility is that these compounds impart a competitive advantage in the CF lung environment as HMAQs produced by *B. cepacia* exhibit antifungal activity [60], so it is feasible that the *hmqABCDEFG* operon of *B. pseudomallei* produces similarly potent compounds that can inhibit fungal species from establishing residence in the CF lung. The convergent upregulation of *hhqA–hhqG* in the *B. pseudomallei* CF isolates points to a putative role for AHQ-based compounds in *B. pseudomallei* signalling, immune evasion or competition in the CF lung. More work is needed to elucidate the myriad functions of AHQ compounds in *B. pseudomallei*, and particularly their role in promoting bacterial persistence in the CF airways.

Of the two convergently downregulated loci, only one, *BPSS0351*, has an assigned function, although little is known about the role of this gene and its product in *B. pseudomallei*. This gene encodes CueR (3.4-fold; 11×), a MerR family copper response regulator that is highly sensitive to the presence of copper (Cu) and which regulates the transcription of genes that protect against toxic metal ion concentrations [61, 62]. Cu has a long history as an effective antimicrobial agent due its ability to generate reactive oxygen species, with Cu accumulation in the mammalian host purported to act as an innate immune defence mechanism to restrict pathogen growth [63]. Thus, downregulation of *cueR* in the latter CF isolates may represent a mechanism for mitigating Cu toxicity in the host, similarly to *Escherichia coli* [62]. However, there are contradictory reports as to whether Cu levels are elevated in CF sputa [64, 65], and the artificial sputum growth medium does not appear to contain elevated Cu levels [18]. CueR regulates the Cu/silver ATPase CopA and the multicopper oxidase CueO enzymes in *E. coli*, which correspond to *BPSS0224* and *BPSL0897* in *B. pseudomallei* K96243, respectively; however, neither of these genes showed DE in any of the patient pairs. The absence of concomitant increased expression of *cueO*, which converts periplasmic Cu+ to less toxic Cu2+ *in* vivo [38], suggests that other enigmatic pressures are responsible for decreasing *cueR* expression in latter CF isolates. The biological role of these other factors requires further exploration.

We recognise that there are limitations to our study. Growth conditions are known to be an important consideration for mRNA-based investigations due to the alteration of the transcriptome when isolates are grown under different environments or media components [66]. Although our *in vitro* conditions do not completely mimic the conditions seen in the CF lung, the artificial sputum medium is designed to reflect the nutrient conditions of this environment [18], and our shaking parameters provided a robust way of measuring cellular growth over time while avoiding non-uniform cellular growth, which ensured harvest of *B. pseudomallei* cultures at the same growth phase (Fig. S1). Additionally, the use of isogenic strain pairs with genomic data [14] enabled us to comprehensively assess the effects of transcriptional adaptation to the CF lung compared with their underlying genetic variants. Our conditions provided transcriptomic data that were consistent with expected expression differences based on genome-wide alterations. Other studies have used artificial sputum media and additional mechanical methods to mimic the CF lung conditions. A rotating wall vessel has been developed to simulate the low fluid shear conditions encountered in CF mucus due to pathological effects of *CFTR* dysfunction on mucociliary clearance [67], with CF-derived *P. aeruginosa* isolates demonstrating transcriptional differences depending on shear conditions [19]. The culturing methods for bacterial RNA-seq are a critical consideration in experimental design as they can affect transcriptomic profiles, and the impact of conditions should be considered when comparing transcriptional differences between studies. Another shortcoming is that we only examined five patient pairs due to the relative paucity of melioidosis CF cases worldwide, and only two isolates from each patient due to limited bacterial colony selection and storage at the time of sputum collection and processing. Deeper sampling efforts across a greater number of melioidosis CF patients would be needed to provide greater confidence in our convergent adaptation findings and would allow a more thorough understanding of *B. pseudomallei* population dynamics and diversity to be attained. Nevertheless, the findings from our study provide important new insights into *B. pseudomallei* evolution in the CF airways, with many, although not complete, parallels with the common CF pathogens, *P. aeruginosa* and *B. cepacia* complex species.

## Data bibliography

1. K96243. Holden MT, Titball RW, Peacock SJ, Cerdeño-Tárraga AM, Atkins T, Crossman LC, Pitt T, Churcher C, Mungall K, Bentley SD, Sebaihia M, Thomson NR, Bason N, Beacham IR, Brooks K, Brown KA, Brown NF, Challis GL, Cherevach I, Chillingworth T, Cronin A, Crossett B, Davis P, DeShazer D, Feltwell T, Fraser A, Hance Z, Hauser H, Holroyd S, Jagels K, Keith KE, Maddison M, Moule S, Price C, Quail MA, Rabbinowitsch E, Rutherford K, Sanders M, Simmonds M, Songsivilai S, Stevens K, Tumapa S, Vesaratchavest M, Whitehead S, Yeats C, Barrell BG, Oyston PC, Parkhill J. NCBI GenBank accession numbers NC_006350.1 and NC_006351.1 (2004).
2. MSHR5651 (CF6). Viberg LT, Price EP, Kidd TJ, Bell SC, Currie BJ, Sarovich DS. NCBI GenBank accession number JYBG00000000 (2015).
3. MSHR8436 (CF8). Viberg LT, Price EP, Kidd TJ, Bell SC, Currie BJ, Sarovich DS. NCBI GenBank accession number JYBH00000000 (2015).
4. MSHR5662 (CF9). ﻿Viberg LT, Price EP, Kidd TJ, Bell SC, Currie BJ, Sarovich DS. NCBI GenBank accession number JYBI00000000 (2015).
5. MSHR8438 (CF10). Viberg LT, Price EP, Kidd TJ, Bell SC, Currie BJ, Sarovich DS. NCBI GenBank accession number JYBJ00000000 (2015).
6. MSHR8441 (CF11). Viberg LT, Price EP, Kidd TJ, Bell SC, Currie BJ, Sarovich DS. NCBI GenBank accession number JYBK00000000 (2015).

## Supplementary Data

Supplementary File 1

## Supplementary Data

Supplementary File 2

## Supplementary Data

Supplementary File 3

## Supplementary Data

Supplementary File 4
