# Supplementary material for: Transcriptomic analysis of longitudinal Burkholderia pseudomallei infecting the cystic fibrosis lung
Source: Microb Genom. 2018 Jul 10;4(8):e000194. doi: 10.1099/mgen.0.000194 (PMC6159556; doi:10.1099/mgen.0.000194)
Supplement: Supplementary File 4 [file mgen-5-194-s004.pdf]

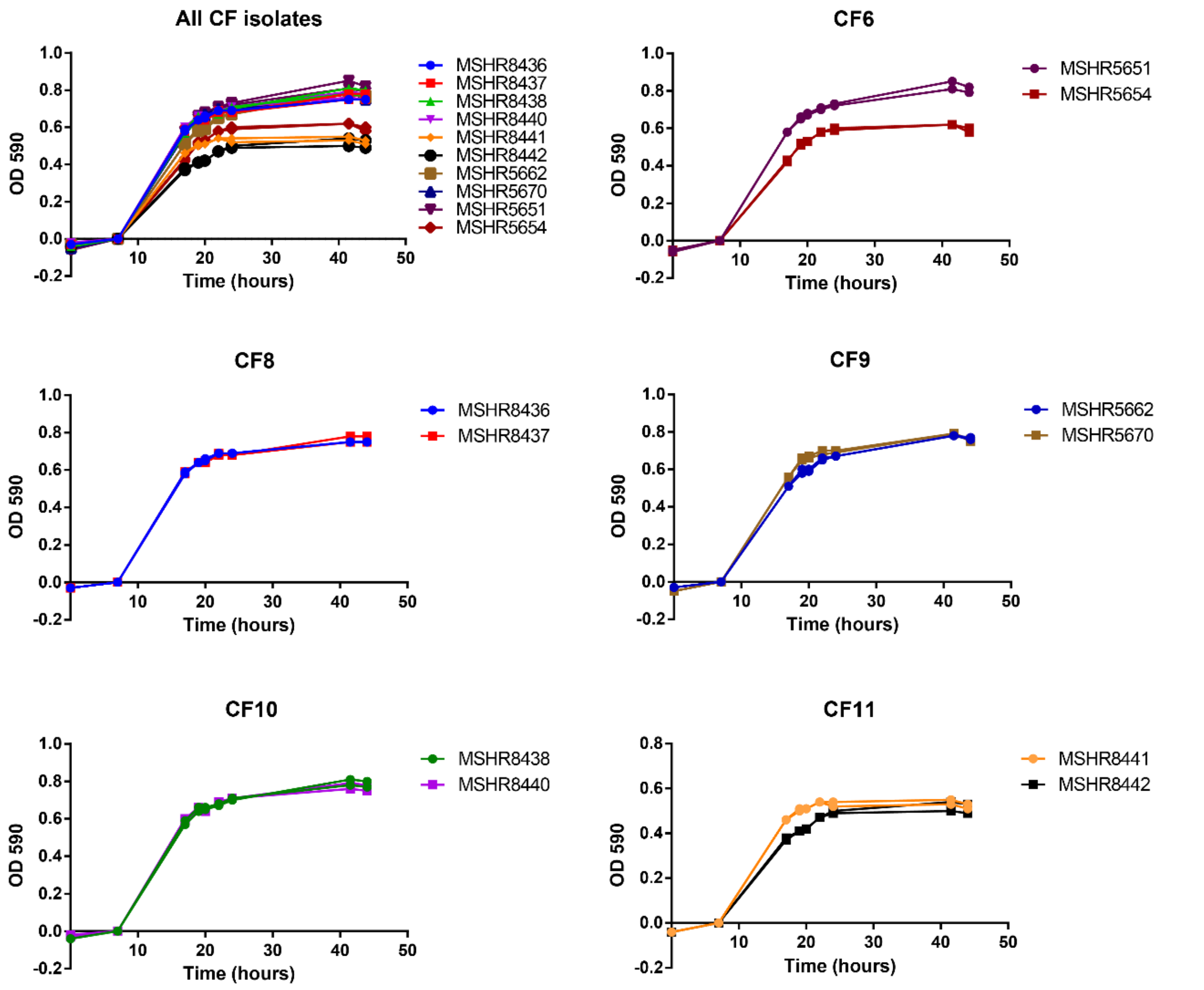

Figure S1. Growth curve analysis of longitudinal *Burkholderia pseudomallei* isolates from five Australian cystic fibrosis patients. Two replicates of each culture were tested. Approximately 10^5 colony-forming units were inoculated into a sterile artificial sputum medium mimicking CF sputum and shaken at 200 rpm for 44h. These data were used to determine the optimal time for harvesting cells at late-log phase for RNA-seq.
